# Space-Time scan of tuberculosis indicators in Northeast Brazil: an ecological and time-series study over 20 years (2001-2020)

**DOI:** 10.1186/s12889-025-24307-6

**Published:** 2025-11-24

**Authors:** Mariana do Rosário Souza, Vinícius Barbosa dos Santos Sales, Gleidson Felipe Hilario de Jesus, Lucas Almeida Andrade, Ana Cristina de Oliveira Costa, Carlos Dornels Freire de Souza, Álvaro Francisco Lopes de Sousa, Patrícia P. da S. Picelli Sanches, Allan Dantas dos Santos, Emerson Lucas Silva Camargo, Shirley V. M. Almeida Lima, Karina Conceição G. M. Araújo, Isabel Amélia Costa Mendes, Márcio Bezerra-Santos

**Affiliations:** 1 Graduate Program in Health Sciences, Universidade Federal de Sergipe, São Cristóvão, Sergipe Brasil; 2 Graduate Program in Public Health, Instituto René Rachou, Belo Horizonte, Belo Horizonte, Minas Gerais Brasil; 3 Universidade Federal do Vale do São Francisco, São Paulo, Brasil; 4 Instituto de Ensino e Pesquisa, Hospital Sírio-Libanês, Rua Dona Adma Jafet, 91, Bela Vista, São Paulo, CEP: 01308-050 Brasil; 5 Comprehensive Health Research Center (CHRC), NOVA National School of Public Health, Nova University of Lisbon, Lisbon, Portugal; 6 Universidade Federal de Sergipe, Aracaju, Sergipe Brasil; 7 Ribeirão Preto College of Nursing, Universidade de São Paulo, Ribeirao Preto, Brasil; 8 Complex of Medical and Nursing Sciences, Federal University of Alagoas, Arapiraca, Alagoas Brasil

**Keywords:** Tuberculosis, Epidemiological and clinical indicators, Spatial analyses, Health services, Northeast, Brazil

## Abstract

**Background:**

Tuberculosis (TB) is a persistent infectious disease caused by the *Mycobacterium tuberculosis* bacillus. Despite being preventable and treatable, TB remains a significant global public health challenge, particularly affecting vulnerable populations such people deprived of liberty, homeless individuals, and those living with HIV/AIDS. In Brazil, regional disparities may influence the prevalence and mortality rates of TB, highlighting the need for comprehensive epidemiological assessments to inform targeted interventions.

**Objectives:**

This study aimed to evaluate the clinical and epidemiological indicators, temporal trends, and spatial distribution of pulmonary tuberculosis (PTB) in the Northeast region of Brazil.

**Methods:**

An ecological and time-series study spanning two decades was conducted using PTB indicators from all nine Federative Units in the Northeast region of Brazil. Temporal trends were assessed using segmented linear regression models, while spatial analysis employed global and local Moran indices to detect clustering patterns. Space-time scanning statistics were also utilized to identify high-risk clusters of PTB cases.

**Results:**

Between 2001 and 2020, a total of 426,110 cases of PTB were reported in the Brazilian Northeast, with a predominant occurrence among individuals aged 20–39 years, non-white individuals, and males. The region exhibited an incidence coefficient of 35.94 cases and a mortality rate of 1.15 per 100,000 inhabitants. Pernambuco emerged with the highest incidence and mortality rates, followed by Sergipe, which also reported the highest proportion of treatment success (72.05%) and interruption or failure (12.77%). While the detection rate remained stable over time, there was a concerning upward trend in mortality rates across all Federative Units, with an average annual percentage change of 17.4%.

**Conclusions:**

The study revealed a heterogeneous distribution of PTB across the Northeast region of Brazil, with notable high-risk clusters identified primarily in Bahia, Ceará, Maranhão, and coastal areas of Alagoas and Pernambuco. The stability in PTB incidence coupled with rising mortality rates and declining cure proportions underscores significant challenges in TB control efforts within the region. These findings underscore the urgent need for targeted interventions and strengthened healthcare systems to achieve Brazil’s goals outlined in the “End TB strategy” endorsed by the WHO.

## Introduction

Tuberculosis (TB) is a severe infectious disease with a chronic course, caused by intracellular bacilli belonging to the species *Mycobacterium tuberculosis*. The presentation of tuberculosis in the pulmonary form (PTB), in addition to being more frequent, is also the most relevant for public health, since it is this form, especially the bacilliferous form, that is responsible for maintaining the chain of transmission of the disease. According to the World Health Organization (WHO), 10.6 million people worldwide fall ill with TB annually. Despite being preventable and curable, 1.6 million people die from it each year, making it the 13th leading cause of death and the second leading cause of infectious death after COVID-19 [1, 2].

Furthermore, the WHO recommends timely and early diagnosis of PTB, with immediate initiation of treatment, in order to reduce the risk of progression with clinical complications and transmission of the bacillus. In Brazil, various measures and strategies are implemented for TB prevention and control. However, the incidence coefficients continue to be significant. In 2020, the rate was 36.3/100,000 inhabitants (78,057 cases), highlighting the urgency to intensify and develop new strategies for controlling this endemic disease [1–3]. Additionally, the COVID-19 pandemic has negatively impacted TB diagnoses in Brazil. A previous study conducted by our group demonstrated an 8.3% reduction in TB diagnoses in Brazil during 2020. These findings imply new challenges for the country to achieve the TB elimination goal by 2030 [4].

The prevalence of TB is directly related to the living conditions and quality of life of populations. Remarkably, the disease affects more severely those living in greater socioeconomic vulnerability. In a country with continental dimensions like Brazil and with climatic and economic differences among regions, it is expected that the poorest regions would be the most affected by the disease. According to Brito and colleagues [5], the Northeast presents consistent evidence regarding the maintenance of the TB transmission chain.

Understanding the panorama and spatiotemporal dynamics of morbidity and mortality from TB in this region can greatly contribute to health services for planning, investments, and improvements in TB diagnosis, control, and prevention programs. Therefore, this study aims to analyze the clinical-epidemiological profile and spatial and spatiotemporal distribution of pulmonary tuberculosis cases in the Northeast region of Brazil, from 2001 to 2020.

## Materials and methods

### Study design

This study adopts an epidemiological approach, employing ecological and time-series analyses spanning from 2001 to 2020. Spatial analysis techniques are utilized, focusing on cases of pulmonary tuberculosis documented in the Notifiable Diseases Information System (NDIS). Access to this system is public and facilitated through the official websites of the Brazilian Ministry of Health, with no individual identification.

### Study area

**Fig. 1:**
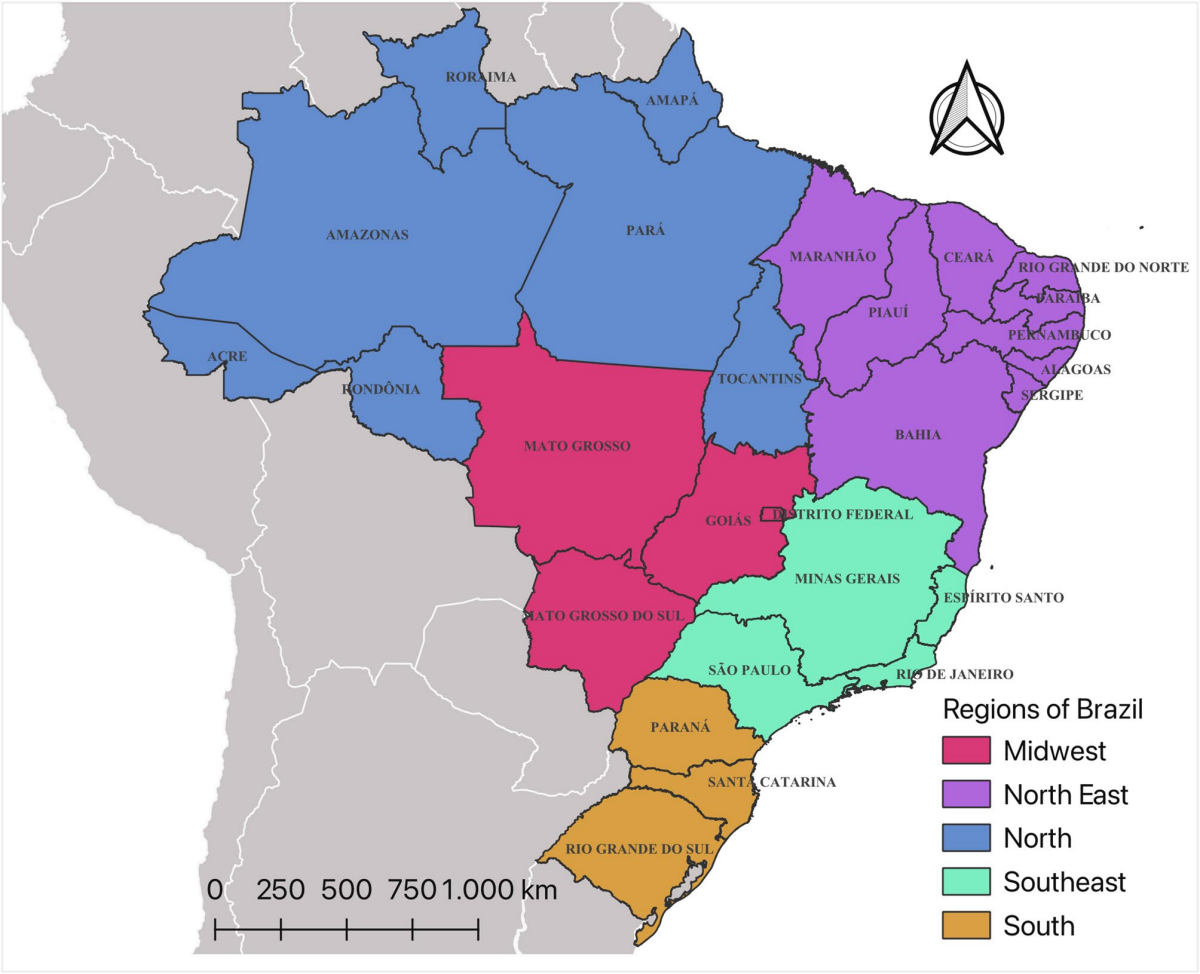
The geopolitical regions and Federative Units of Brazil. Source: own authorship.

The Northeast region constitutes one of Brazil’s five geopolitical divisions, encompassing an area of 1,554,257 km2 (latitude: 01º02’30” N/18º20’07” S; longitude: 34º47’30”/48º45’24” W). This area represents 18.2% of the country’s total landmass and is divided into nine federative units (UF): Alagoas (AL), Bahia (BA), Ceará (CE), Maranhão (MA), Paraíba (PB), Pernambuco (PE), Piauí (PI), Rio Grande do Norte (RN), and Sergipe (SE). The region is estimated to have a population of 55,389,382 inhabitants (as of 2022), with a Gini Index of 0.6277 (2010), and a Human Development Index (HDI) of 0.659. (See Fig. 1)

### Variables and indicators

#### Sociodemographic Variables

The following sociodemographic and clinical-epidemiological characteristics were gathered to delineate the epidemiological profile of pulmonary tuberculosis (PTB) cases in the Brazilian Northeast:

- Age group (0-4 years; 5-9 years; 10-19 years; 20-39 years; 40-59 years; 60 years or older)
- Race/ethnicity (white, black, mixed race, yellow, Indigenous, unknown/missing)
- Gender (unknown/missing, male, female)
- Residential area (urban, rural, peri-urban, unknown/missing)
- Education level (illiterate, incomplete primary education (1st to 4th grade), complete primary education (4th grade), incomplete lower secondary education (5th to 8th grade), complete lower secondary education, incomplete upper secondary education, complete upper secondary education, incomplete higher education, complete higher education, unknown/missing, not applicable)
- Type of entry (new case, relapse, readmission after interruption treatment, transfer, post-mortem, unknown/missing)
- Outcome (unknown/missing, cure, interruption treatment, TB-related death, death from other causes, transfer, Drug-Resistant TB, treatment change, treatment failure, primary interruption treatment)

### Data source

Data regarding PTB cases were provided by the Secretariat of Health and Environmental Surveillance of the Ministry of Health (SVSA/MS) through the databases of the Notifiable Diseases Information System (NDIS). These data are publicly accessible and can be obtained from the website of the Department of Health Informatics of the Unified Health System (DATASUS). NDIS is fed by standardized forms across the Brazilian territory.

Population estimates, per year, for the region and its nine federated units were also sourced from DATASUS. With this information, the clinical-epidemiological profile of PTB cases was analyzed, yielding: Incidence coefficient; Mortality coefficient; Treatment success; and interruption treatment.

The cartographic mesh is provided by another Brazilian public entity, the Brazilian Institute of Geography and Statistics (IBGE). Thus, digital cartographic grids, in shapefile format, obtained from the Geographic Projection System of latitude/longitude (Geodetic Reference System, SIRGAS 2000) were utilized.

## Data processing and analysis

### Time trend analysis

Temporal trend analysis was executed utilizing the joinpoint regression model via Joinpoint Program version 4.7.0.0. Clinical-epidemiological characteristics were regarded as dependent variables, whereas the years of the time series were independent variables. To delineate and quantify trends, the average annual percentage change (AAPC) and their corresponding confidence intervals (95% CI) were computed. Consequently, time series may manifest increasing, decreasing, or stable trends. A positive AAPC value with *p* < 0.05 denotes a significant increasing trend; conversely, a negative AAPC value with *p* < 0.05 signifies a significant decreasing trend; ultimately, non-significant trends are designated as stable, irrespective of the AAPC values [6].

#### Distribution and spacial analysis of data

The spatial distribution maps were created using the QGIS program, version 3.34.0, and analyzed in TerraView. For this purpose, the cartographic base of the Northeast region, divided by Federative Units and municipalities, provided by IBGE (2020), was utilized. Spatial smoothing was performed using the local empirical Bayesian estimator for the thematic maps of PTB and mortality. To stratify the incidence coefficient, the WHO Global Tuberculosis Report was consulted, delineating the ranges as follows: 0–9.9/100,000 population; 10–19/100,000 population; 20–49/100,000 population; 50–124/100,000 population; 125–299/100,000 population [7].

Spatial autocorrelation of the incidence and mortality rates was assessed by computing the Local Moran’s Index to determine whether the spatial distribution of PTB and deaths is random or not. Subsequently, a scatter plot was generated, dividing the municipalities into the following spatial quadrants: Q1 (high/high) and Q2 (low/low), indicating municipalities with similar values to their neighbors, representing areas of positive spatial association; and Q3 (high/low) and Q4 (low/high) with differing values representing transition areas with negative spatial association aggregates [8, 9].

#### Space-time scanning statistics

Space-time scanning statistics were employed to pinpoint high-risk clusters or space-time clusters for PTB cases and mortality in the Northeast region, utilizing the discrete Poisson probability distribution model. The null hypothesis (H0) posits that the anticipated number of PTB cases and deaths in each area is proportional to its population size and mirrors a constant risk. Conversely, the alternative hypothesis (HA) suggests that the observed variables in the study surpass the anticipated number of deaths derived from the null model.

The primary cluster and secondary clusters were identified using the likelihood ratio test (LLR) and depicted in thematic maps. Relative risks (RR) were also computed for PTB cases and mortality, taking into account each municipality in the Northeast region and clusters in relation to their neighbors. Ultimately, results with a p-value < 0.05, utilizing 999 Monte Carlo simulations, and with a confidence interval (95% CI) that does not intersect zero were deemed significant.

## Results

Between 2001 and 2020, 426,110 cases of PTB were documented in the Northeast region of Brazil. The Federative Units with the highest proportion of cases were Bahia (*n* = 113,997; 26.75%), Pernambuco (*n* = 91,098; 21.38%), Ceará (*n* = 74,858; 17.57%), and Maranhão (*n* = 48,931; 11.48%).

The clinical-epidemiological characteristics of PTB cases in the Northeast region, as reported in NDIS (Table 1), indicate that the most affected age group is between 20 and 39 years (43.04%), mixed race is described in 54.65% of reported cases, males are predominant (66.04%), and the majority of cases are located in urban areas (62.93%). New cases (79.10%) were the most frequent type of entry.

**Table 1:** Clinical and epidemiological characteristics of pulmonary tuberculosis cases reported in the NDIS in the Northeast region between the years 2001 and 2020

Variables		*n* (%)
Age group	< 4 years	4710 (1.10)
0.04^a^	5–9 years	2427 (0.57)
	10–19 years	31,055 (7.29)
	20–39 years	183,386(43.04)
	40–59 years	137,195(32.20)
	> 60 years	67,157 (15.76)
Race/ethnicity	White	61,831(14.51)
16.82^a^	Black	52,455(12.31)
	Asian	4827(1.13)
	Mixed race	232,861 (54.65)
	Indigenous	2440(0.58)
Gender	Male	281,397(66.04)
0.03^a^	Female	144,611(33.93)
Residential área	Urban	268,145 (62.93)
24.09^a^	Rural	52,637(12.35)
	Peri-urban	2684 (0.63)
Education level	Illiterate	52,100 (12.27)
27.70^a^	Incomplete primary education (1st to 4th grade)	73,073 (17.19)
	Complete primary education (4th grade)	20,792 (4.88)
	Incomplete lower secondary education (5th to 8th grade)	71,554 (16.79)
	Complete lower secondary education	16,037 (3.76)
	Incomplete upper secondary education	30,786 (7.22)
	Complete upper secondary education	24,876 (5.84)
	Incomplete higher education	3256 (0.76)
	Complete higher education	8815 (2.07)
	Not applicable	6830 (1.60)
Type of entry	New case	335,179 (78.66)
0.00^a^	Relapse	28,180 (6.61)
	Readmission after interruption treatment	28,285 (6.64)
	Unknown	11,358 (2.66)
	Transfer	21,849 (5.13)
	Post-mortem	1253(0.30)
Outcome	Treatment success	286,451 (67.22)
3.75^a^	Interruption treatment	47,829 (11.22)
	Tuberculosis-related death	11,470 (2.70)
	Death from other causes	16,771 (3.93)
	Transfer	43,609 (10.23)
	Drug-resistant tuberculosis	2533 (0.60)
	Treatment change	712 (0.17)
	Treatment failure	141 (0.03)
	Primary interruption treatment	592 (0.14)

^a^Missing value percentual

The highest incidence coefficient of PTB belongs to the Federative Unit of Pernambuco, 55.66 per 100,000 inhabitants (Table 2), as well as the highest mortality rate (40.50 per 100,000 inhabitants). Sergipe has the highest proportion of treatment success and interruption treatment, 72.05% and 12.77%, respectively.

**Table 2:** Incidence coefficient, mortality coefficient, treatment success, and interruption treatment of pulmonary tuberculosis cases in the Northeast region, by federative unit, reported in NDIS, between the years 2001 to 2020

Variables	Federative units	Morbimortality PTB
Incidence coefficient^a^	**Northeast**	**35.94**
	Alagoas	39.65
	Bahia	43.82
	Ceará	46.80
	Maranhão	38.21
	Paraíba	33.60
	Pernambuco	55.66
	Piauí	30.21
	Rio Grande do Norte	36.09
	Sergipe	31.30
Mortality coefficient^a^	**Northeast**	**1.15**
	Alagoas	1.39
	Bahia	0.97
	Ceará	0.99
	Maranhão	0.92
	Paraíba	0.79
	Pernambuco	2.03
	Piauí	0.71
	Rio Grande do Norte	1.17
	Sergipe	0.90
Treatment success	**Northeast**	**66.92%**
	Alagoas	67.63%
	Bahia	65.89%
	Ceará	68.58%
	Maranhão	70.74%
	Paraíba	64.18%
	Pernambuco	63.83%
	Piauí	71.64%
	Rio Grande do Norte	67.04%
	Sergipe	72.05%
Interruption treatment	**Northeast**	**11.01%**
	Alagoas	12.02%
	Bahia	9.03%
	Ceará	11.83%
	Maranhão	11.33%
	Paraíba	12.17%
	Pernambuco	12.29%
	Piauí	5.32%
	Rio Grande do Norte	10.98%
	Sergipe	12.77%

^a^The result should be read per 100,000 inhabitants

The temporal trend of the PTB incidence coefficient is decreasing in the Federative Units of Maranhão, Piauí, Ceará, and Bahia, but increasing in Pernambuco (Table 3). The temporal trend of the PTB mortality coefficient is increasing in the Northeast region (17.4% per year). The temporal trend of treatment interruption is increasing in the Federative Units of Ceará and Bahia (4.0% and 1.2% per year, respectively) and decreasing in Rio Grande do Norte (1.2% per year). The temporal trend of the treatment success in the Northeast region is decreasing (0.7% per year).

**Table 3:** Temporal trend of pulmonary tuberculosis morbimortality in the Northeast region reported in NDIS, between the years 2001 and 2020

Variables	Federative Unit	AAPC**	95% CI	Trend
**Incidence coefficient**	**Northeast**	**−1.7**	**−3.7 ~ 0.3**	**Stable**
	Maranhão	−1.8*	−3.0~−0.5	Decreasing
	Piauí	−3.5*	−4.5~−2.5	Decreasing
	Ceará	−1.2*	−1.7~−0.8	Decreasing
	Rio Grande do Norte	0.4	−1.0 ~ 1.9	Stable
	Paraíba	−0.6	−1.3 ~ 0.1	Stable
	Pernambuco	0.8*	0.2 ~ 1.4	Increasing
	Alagoas	−2.0	−4.6 ~ 0.6	Stable
	Sergipe	1.3	−0.3 ~ 2.9	Stable
	Bahia	−3.2*	−3.6~−2.8	Decreasing
**Mortality coefficient**	**Northeast**	17.4*	**9.0 ~ 26.4**	**Increasing**
	Maranhão	15.8*	4.3 ~ 28.6	Increasing
	Piauí	45.0*	25.7 ~ 67.2	Increasing
	Ceará	44.1*	31.7~−57.6	Increasing
	Rio Grande do Norte	39.6*	23.5 ~ 57.7	Increasing
	Paraíba	41.0*	28 ~ 55.3	Increasing
	Pernambuco	26.1*	20.1 ~ 32.3	Increasing
	Alagoas	6.0*	1.2 ~ 11.1	Increasing
	Sergipe	42.2*	30.6 ~ 54.9	Increasing
	Bahia	17.0*	9.5 ~ 25.0	Increasing
**Interruption treatment**	**Northeast**	**0.4**	** −0.8 ~ 1.6**	**Stable**
	Maranhão	−0.4	−2.7 ~ 2.0	Stable
	Piauí	1.0	−0.6 ~ 2.6	Stable
	Ceará	4.0*	3.0 ~ 5.0	Increasing
	Rio Grande do Norte	−1.2*	−2.3~−0.2	Decreasing
	Paraíba	−0.7	>−6.2 ~ 5.1	Stable
	Pernambuco	−1.7	−4.3 ~ 1.0	Stable
	Alagoas	−0.5	−1.4 ~ 0.4	Stable
	Sergipe	3.6	−0.2 ~ 7.5	Stable
	Bahia	1.2*	0.7 ~ 1.7	Increasing
**Treatment success**	**Northeast**	−0.7*	** −1.3~−0.1**	**Decreasing**
	Maranhão	−0.2	−0.5 ~ 0.1	Stable
	Piauí	−1.2*	−2.2~−0.2	Decreasing
	Ceará	−0.5	−1.4 ~ 0.3	Stable
	Rio Grande do Norte	−0.6*	−1.0~−0.2	Decreasing
	Paraíba	−2.1*	−3.1~−1.2	Decreasing
	Pernambuco	−0.2	−1.0 ~ 0.6	Stable
	Alagoas	−1.4*	−2.1~−0.7	Decreasing
	Sergipe	−0.6*	−0.9~−0.2	Decreasing
	Bahia	−0.9	−1.7 ~ 0.0	Stable

*p < 0.05

** Average Annual Percentage Change

**Fig. 2:**
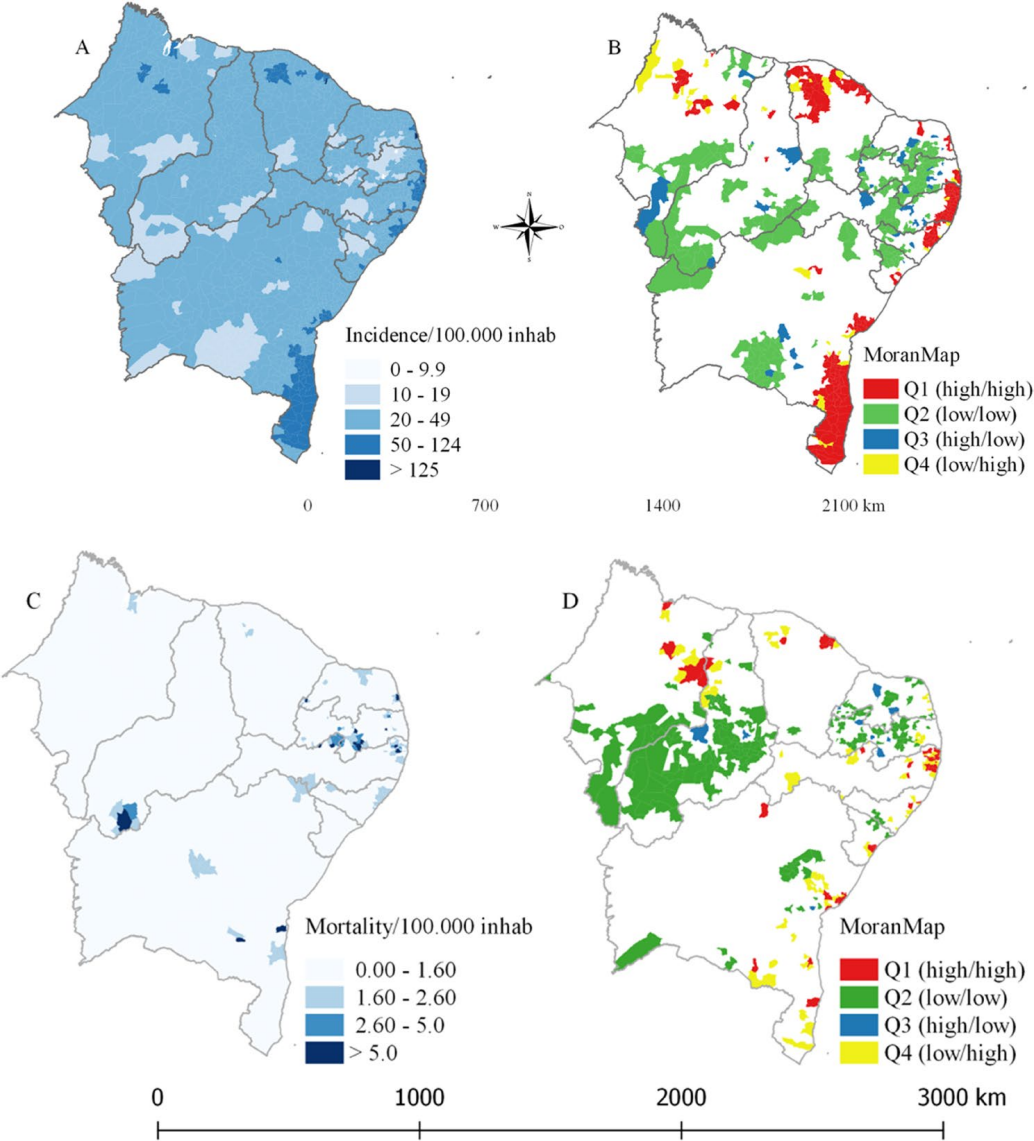
Spatial distribution of PTB incidence coefficient and mortality coefficient in the Northeast region between the years 2001 to 2020. **A**. Local Empirical Bayesian of incidence coefficient. **B** MoranMap of incidence coefficient. **C** Local Empirical Bayesian of mortality coefficient. **D** MoranMap of mortality coefficient

The Local Empirical Bayesian estimator of PTB incidence is shown in Fig. 2A. Incidences exceeding 125 cases per 100,000 population are observed in 4 municipalities: Ilha de Itamaracá (PE), Itapissumã (PE), Itaitinga (CE), and Nísia Floresta (RN). Another 133 municipalities fall within the range of 50 to 124 cases per 100,000 inhabitants. The Local Moran was 0.4087 with a p-value of 0.01, indicating positive spatial autocorrelation. The MoranMap of PTB incidence (Fig. 2B) shows 220 municipalities in quadrant Q1 (high/high).

Using the Local Empirical Bayesian estimator of TBP mortality, it is possible to visualize 18 municipalities with a coefficient above 5 deaths per 100,000 population (Fig. 2C). The Moran’s Index for mortality was 0.4088 with a p-value < 0.05, highlighting 47 municipalities classified in Q1 (Fig. 2D).

**Fig. 3:**
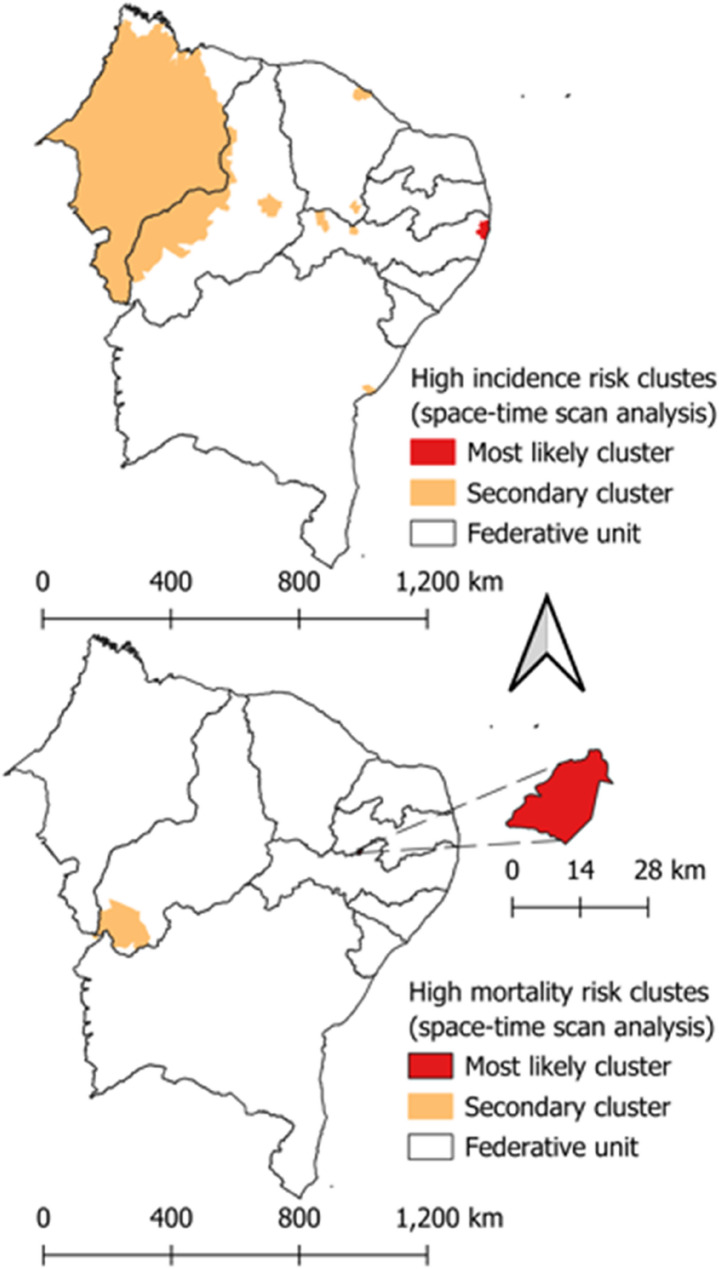
Space-time scanning map. **A** Space-time scanning map of the incidence coefficient of PTB in the Northeast region between the years 2001 and 2020. **B** Space-time scanning map of the mortality coefficient of PTB in the Northeast region between the years 2001 and 2020. Source: own authorship

According to the space-time scanning for PTB occurrence (Fig. 3A), the primary cluster includes 10 municipalities in the Federative Unit of Pernambuco (Olinda, Paulista, Recife, Abreu e Lima, Camaragibe, Igarassu, São Lourenço da Mata, Jaboatão dos Guararapes, Itapissuma, and Ilha de Itamaracá), and the relative risk of illness (RRI) is 2.38 times higher when compared to other municipalities in the Northeast. Secondary clusters are also observed in the Federative Units of BA, CE, PE, MA, and PI, with emphasis on the cluster involving 208 municipalities in PI and MA (RR = 1.21).

According to the space-time scanning for TB mortality (Fig. 3B), the primary cluster is located in the municipality of Quixabá, Federative Unit of PE, and the relative risk of death (RRM) is 1,584.20 times higher than in other municipalities in the Northeast. A secondary cluster is observed in the Federative Unit of PI and involves 7 cities (RRM = 4.06).

## Discussion

Governmental measures implemented to combat PTB nationwide have not been sufficient to contain the disease’s progression in the Brazilian Northeast. The region faces a severe epidemiological situation concerning the global objectives outlined by the WHO’s “End TB Strategy,” which aims to reduce the TB incidence coefficient by 80% by 2030 [10]. In this regard, the Brazilian Northeast alone registered 426,110 new cases of PTB over a 20-year period (2001–2020).

The indices examined in this study underscore the rise in disease incidence above the national average in most Federative Units of the region (95.94/100,000 population), alongside an increasing temporal trend in mortality across all Federative Units, while maintaining a stable temporal trend in incidence. Furthermore, the decline in the proportion of cured patients (0.7% per year) highlights the fragility in treatment adherence, which should be tailored to ensure better care quality and address TB as a public health concern [11].

In 2012, the Parliamentary Front to Fight TB was established, along with the Brazilian Network of Federative Unit Committees, aimed at facilitating the approval of public policies aimed at reducing disease cases. However, these committees were not implemented in the Federative Units of Sergipe, Alagoas, and Rio Grande do Norte, hindering the integration of governmental and civil society entities [12, 13].

The study revealed that adults aged 20 to 59 account for over 70% of PTB cases in the Northeast, a demographic group considered economically active, which could impact the country’s financial health due to symptoms and the initial isolation required affecting work capacity [14].

Another study conducted in the Federative Unit of Sergipe between 2001 and 2016 observed a growing trend of TB cases in patients under 20 years old [15]. Our research observed the occurrence of the disease in children under 15 years old, as was also noted by Brito et al. [5].

Regarding the epidemiological profile of TB in the Northeast, individuals with non-white skin color, low education levels, and male gender predominate. According to the Ministry of Health [3], men aged 20 to 34 are 2.8 times more likely to develop PTB than women in the same age group, a trend found in other regions of the country [16–19]. It is noteworthy that this population segment is more exposed to risk factors associated with the disease, such as the abusive use of legal and illegal drugs, as well as a higher incidence of HIV/AIDS infection. Moreover, men seek healthcare services less frequently, hindering early diagnosis and adequate PTB control [20, 21].

Additionally, there is a higher incidence of PTB in urban areas, which reinforces the high transmissibility of the disease in locations with higher population density, overcrowded conditions, and poor housing. According to Zhang et al. (2022) [22], factors such as income, unemployment rate, educational level, medical resources, population density, and dietary structure are closely related to TB incidence.

For PTB control, a minimum Treatment success of 90% is required, but the Brazilian Northeast presented a rate of 66.92%, with the best proportion among Federative Units being in Sergipe (72.05%), still far below the expected [2]. Rabahi et al. (2017) [23] indicate that changes in PTB treatment in 2009 failed to contain the decrease in Treatment success, the increase in treatment interruption treatment rates, and the increase in Multidrug-Resistant Tuberculosis (MDR-TB) rates, being associated with increased mortality.

According to our study, between the years 2001 and 2020, the Brazilian Northeast experienced an increase of 17.34% per year in PTB mortality and a reduction in the cure proportion (−0.7% per year), alongside a stable trend in treatment interruption treatment proportion. An alarming point is that the mortality rate in the region was decreasing until 2005 [24].

We emphasize that the elimination of the disease as a public health problem also depends on the engagement and training of healthcare professionals to strengthen treatment adherence. Treatment interruption treatment can have significant consequences for the patient and the community, as it maintains the transmission chain and increases the risk of drug resistance and TB-related deaths [1, 25].

The WHO stipulates that 5% of cases end in interruption treatment; however, no Federative Unit in the region reached the target considering the average of the 20 years analyzed. Another point worth noting is the increasing temporal trend of interruption treatment in the Federative Units of BA and CE. A study conducted in the capital of CE, Fortaleza, reveals that the likelihood of interruption treatment showed an increasing trend over the years, with the likelihood of treatment interruption treatment being 71% higher in 2014 than in 2007 [26]. As interruption treatment demonstrates weaknesses in adherence and healthcare services, professionals comprising the Family Health Team (FHT) must be able to develop personalized treatments that involve the patient, as attitudes have the potential to improve or compromise outcomes [11, 27].

Regarding the findings of the spatial analysis of PTB cases in the Northeast, incidence clusters are in coastal regions and near the capitals, reinforcing the impact of spatial clusters with high population density as a determining factor in *Mycobacterium tuberculosis* transmission. However, mortality clusters are displaced to the interior of the Federative Units. The Moran Index for mortality was 0.4088, that is, the distribution of cases does not occur in a verified manner. In the literature, mortality is strongly associated with social determinants of health and difficulty in accessing services [28–30].

The WHO Commission on Social Determinants of Health (2010) [31] reinforces the importance of identifying social determinants for health equity and progress in countries. In the Central-West region of Brazil, the risk of mortality from tuberculosis is a problem associated with social determinants [28]. In another study carried out in RN, mortality from PTB was associated with a high index of social vulnerability and difficulty in accessing services [29].

The space-time scanning indicates vast areas with a high risk of illness, but these do not intersect with municipalities with the highest risk of death. It is also noticeable that there are higher chances of death the farther away from the Federative Unit capital. Thus, public policies should be directed towards diagnostic services with timely laboratory results and access to drug treatment. Primary Health Care professionals are responsible for welcoming patients with a stigmatized disease and developing strategies for treatment adherence, including the use of Directly Observed Treatment and Singular Therapeutic Project.

The conduct of this study was of great importance in elucidating the serious public health situation of pulmonary tuberculosis (PTB) in the Brazilian Northeast. In this scenario, it is questionable whether there are effective public policies to control the increase in deaths and the decline in treatment successs. The analysis by municipality geographically exposed the most vulnerable areas for illness and death from the disease. Thus, we allow the financial resources for TB control to be directed to priority areas.

### Limitations

Despite the comprehensive analysis conducted in this study, several limitations are worth noting. Firstly, the reliance on data from the Brazilian Notifiable Diseases Information System (NDIS) may introduce biases due to underreporting or misclassification of PTB cases, potentially leading to an underestimation of disease burden. Additionally, the use of secondary data limits the availability of detailed clinical information and may overlook certain confounding factors that could influence disease incidence and outcomes, such as comorbidities or socioeconomic status. Furthermore, the study’s focus on the Northeast region of Brazil may limit the generalizability of findings to other regions or countries with different healthcare systems, population demographics, and TB control strategies. Therefore, caution should be exercised when extrapolating these findings to other settings.

## Conclusion

This study sheds light on the persistent challenges faced in controlling pulmonary tuberculosis (PTB) in the Brazilian Northeast region. Despite governmental efforts and initiatives, the region continues to struggle with high PTB incidence and mortality rates, along with declining cure proportions and increasing trends in treatment interruption treatment. These findings underscore the urgent need for targeted interventions and strengthened healthcare systems to address the complex socioeconomic and healthcare access barriers contributing to PTB transmission and outcomes. Moreover, the identification of spatial clusters of PTB cases highlights the importance of tailored public health strategies to address local transmission dynamics effectively. Moving forward, collaborative efforts between policymakers, healthcare professionals, and community stakeholders are essential to achieve the ambitious global targets for PTB control and ultimately alleviate the burden of this disease on affected populations in the Northeast region and beyond.

## Data Availability

All data is provided within the manuscript or supplementary information files.
